# Long-term Survival of Patients with Cholangiolocellular Carcinoma After Curative Hepatectomy

**DOI:** 10.1245/s10434-014-3582-0

**Published:** 2014-03-17

**Authors:** Shun-ichi Ariizumi, Yoshihito Kotera, Satoshi Katagiri, Masayuki Nakano, Yasuni Nakanuma, Akiko Saito, Masakazu Yamamoto

**Affiliations:** 1 Department of Surgery, Institute of Gastroenterology, Tokyo Women’s Medical University, Kawada 8-1, Shinjuku-ku, Tokyo, 162-8666 Japan; 2 Division of Pathology, Ofuna Chuo Hospital, Kamakura, Kanagawa Japan; 3 Department of Human Pathology, Kanazawa University Graduate School of Medicine, Kanazawa, Japan; 4 Department of Internal Medicine, Institute of Gastroenterology, Tokyo Women’s Medical University, Tokyo, Japan

## Abstract

**Background:**

Cholangiolocellular carcinoma (CoCC) has distinct pathological characteristics, and CoCC is considered to originate from hepatic progenitor or stem cells. However, the surgical outcome of CoCC has not been clarified in detail.

**Methods:**

We retrospectively studied 275 patients with intrahepatic cholangiocarcinoma (ICC) who underwent hepatectomy between 1990 and 2011. Surgical outcomes were compared between 29 patients with CoCC and 130 patients with mass-forming (MF) type ICC since all patients with CoCC showed MF type on macroscopic findings.

**Results:**

The number of patients with chronic liver disease was significantly higher in the CoCC group than in the ICC group. The number of patients with abnormal levels of CA19-9 was significantly lower in the CoCC group than in the ICC group. Portal vein invasion and intrahepatic metastasis were significantly lower in patients with CoCC group than in the ICC group. In the CoCC group, 15 of 28 patients survived for more than 5 years after curative surgery whereas 15 of 102 patients with ICC survived for more than 5 years after curative surgery. The 5-year survival rate was significantly higher in patients with CoCC (75 %) than in patients with ICC (33 %, *p* = 0.0005). Multivariate analysis showed CoCC, absence of portal vein invasion or hepatic vein invasion, and absence of intrahepatic metastasis to be significant independent prognostic factors for overall survival in patients with MF-type ICC and CoCC.

**Conclusions:**

CoCC is rare, but patients with CoCC had special characteristics with favorable long-term survival due to its less invasive histopathologic characteristics.

## Introduction

Cholangiolocellular carcinoma (CoCC) is a rare type of primary liver cancer. Steiner and Higginson described the distinct pathological characteristics of CoCC, which derives from the cholangioles or canals of Hering.^1^ This tumor was classified as a special type of intrahepatic cholangiocarcinoma (ICC).^2^,^3^ However, as a result of recent advancements in the study and knowledge of hepatic progenitor or stem cells, CoCC is considered to originate from hepatic progenitor or stem cells.^4^–^7^

ICC is a primary liver cancer derived from cholangiocytes in the biliary tree. The biliary tree is divided into the extrahepatic and intrahepatic bile ducts. The hilar bile ducts are lined with cylindrical mucin-producing cholangiocytes. In the liver, large intrahepatic bile ducts (segmental and septal bile ducts) are lined with similar mucin-producing cells, whereas small intrahepatic bile ducts (interlobular bile ducts and ductules) are lined with mucin-negative cuboidal cholangiocytes. The ductules contain hepatic progenitor cells that can differentiate into both hepatocytes and cholangiocytes. Therefore, hepatic progenitor cell derived tumors can display varying hepatocytic and/or cholangiocytic differentiation characteristics within the same tumor. CoCC is considered a hepatic progenitor cell derived tumor.^4^–^7^ Therefore, CoCC exhibits a mass-forming (MF) type tumor at the periphery of the liver and often shows clinical and imaging findings similar to those of hepatocellular carcinoma (HCC). The pathological features and imaging findings of CoCC have been published, but there has been 1 previous report on the surgical outcomes of CoCC.^4^–^13^ Therefore, we evaluated the surgical outcomes of patients with CoCC.

## Patients and Methods

### Patients

Between 1990 and 2011, 274 patients underwent initial hepatic resection for ICC at our institute. A diagnosis of CoCC was made in 29 patients and ICC in 245 patients according to the General Rules for the Clinical and Pathological Study of Primary Liver Cancer.^8^ In 245 patients with ICC, a further diagnosis was made of MF type in 130 patients, periductal infiltrative (PI) type in 24 patients, intraductal growth (IG) type in 15 patients, and MF+PI type in 76 patients based on the macroscopic findings of ICC. We retrospectively studied 29 patients with CoCC and 130 patients with MF-type ICC because all patients showed MF type on macroscopic findings. The patient characteristics are shown in Table 1. Written informed consent was obtained from all patients before hepatectomy. This study was approved by the ethics committee of Tokyo Women’s Medical University.

**Table 1:** Patient characteristics

Characteristics	CoCC (*n* = 29)	ICC (*n* = 130)	*p* value
Sex, male	14 (48 %)	96 (74 %)	0.007
Age, years; median (range)	65 (30–84)	65.5 (26–83)	0.94
Chronic hepatitis or cirrhosis			
Present	22 (76 %)	63 (48 %)	0.0075
HBV	5	16	
HCV	14	36	
HBV+HCV	0	2	
Alcohol	1	8	
NASH	1	1	
Others	1	1	
ICGR_15_, %; median (range)	10.5 (5–52)	9 (1–56)	0.0179
AFP^a^			
Median (range)	8 (1–6826)	5 (1–5854)	0.13
>10 ng/ml	13/27 (48 %)	40/120 (33 %)	0.18
CA19-9^b^			
Median (range)	17 (1–113)	67 (1–12,000,000)	0.47
>37 U/ml	8/27 (30 %)	71/115 (62 %)	0.0025
Arterial CT findings, high density	13/27 (48 %)	13/130 (10 %)	<0.0001
Preoperative diagnosis			
ICC	10 (34 %)	83 (64 %)	0.0064
HCC	16 (55 %)	40 (31 %)	
Metastasis	1 (4 %)	6 (4 %)	
Other	2 (7 %)	81 (1 %)	
Surgical procedure			
Hemihepatectomy or larger	13 (45 %)	81 (62 %)	0.17
Sectionectomy	6 (21 %)	34 (18 %)	
Segmentectomy	10 (34 %)	26 (20 %)	
Lymph node dissection present	11 (38 %)	69 (53 %)	0.14
Bile duct resection present	4 (14 %)	23 (18 %)	0.79
Curative resection, R0	29 (97 %)	102 (78 %)	0.0225
Tumor size, cm; median (range)	3.5 (0.6–13)	6 (1–19)	0.0069
Portal vein invasion, present	15 (52 %)	92 (71 %)	0.0481
Hepatic vein invasion, present	4 (14 %)	26 (20 %)	0.44
Intrahepatic metastasis, present	3 (10 %)	54 (42 %)	0.0015
T classification			
T1	8 (28 %)	19 (15 %)	0.15
T2	20 (69 %)	97 (67 %)	
T3	1 (3 %)	14 (9 %)	
N classification, N1	4 (14 %)	38 (29 %)	0.09
M classification, M1	0	12 (9 %)	0.09
Mucus secretion, present	8 (28 %)	81 (62 %)	0.0007
CK19, present	27 (93 %)	118 (91 %)	0.99
Growth pattern, replacement	24 (83 %)	26 (20 %)	<0.0001
Portal tract within the tumor, present	28 (97 %)	91 (70 %)	0.0017
Ductal plate malformation, present	10 (34 %)	4 (3 %)	<0.0001

TNM classifications were determined according to the American Joint Committee on Cancer (AJCC)/International Union Against Cancer (UICC) TNM Classification and Stage Groups for ICC, 7th ed

*CoCC* cholangiolocellular carcinoma, *ICC* intrahepatic cholangiocarcinoma, *HBV* hepatitis B virus, *HCV* hepatitis C virus, *NASH* nonalcoholic steatohepatitis, *ICGR*_*15*_ indocyanine green retention rate at 15 min, *AFP* alpha-fetoprotein, *CA19*-*9* cancer-associated carbohydrate antigen 19-9, *HCC* hepatocellular carcinoma

^a^147 patients were assessed

^b^142 patients were assessed

### Preoperative Examination

Serum hepatitis B surface antigen, hepatitis C antibody, and indocyanine green retention rate at 15 min were examined preoperatively in all patients. In 29 patients with CoCC, serum alpha-fetoprotein (AFP) and cancer-associated carbohydrate antigen 19-9 (CA19-9) levels were examined preoperatively in 27 patients. Of 29 patients with CoCC, 27 underwent serial abdominal axial single-helical multiphase computed tomography (CT) scans (X vigor; Toshiba, Nasu, Japan) or multidetector helical CT (Aquilion 4 or 16; Toshiba) scans, whereas 2 patients could not undergo the procedure because of iodine allergy.

### Hepatic Resection

All patients underwent hepatectomy, and the choice of resection was made on the basis of the tumor size, tumor location, preoperative diagnosis, and liver function. In patients who were given a diagnosis of HCC preoperatively, hepatectomy without lymph node dissection was performed. On the other hand, in patients who were given a diagnosis of ICC preoperatively, hepatectomy with lymph node dissection (around the hepatoduodenal ligament, the common hepatic artery, or behind the pancreas head) was performed. Extrahepatic bile duct resection and reconstruction was performed when either HCC or ICC involved the bile duct in the perihilar region. Surgical procedures are shown in Table 1. The terminology of liver resection was determined according to the Terminology Committee of the International Hepato-Pancreato-Biliary Association of 2000.^14^

### Pathological Examination

**Fig. 1:**
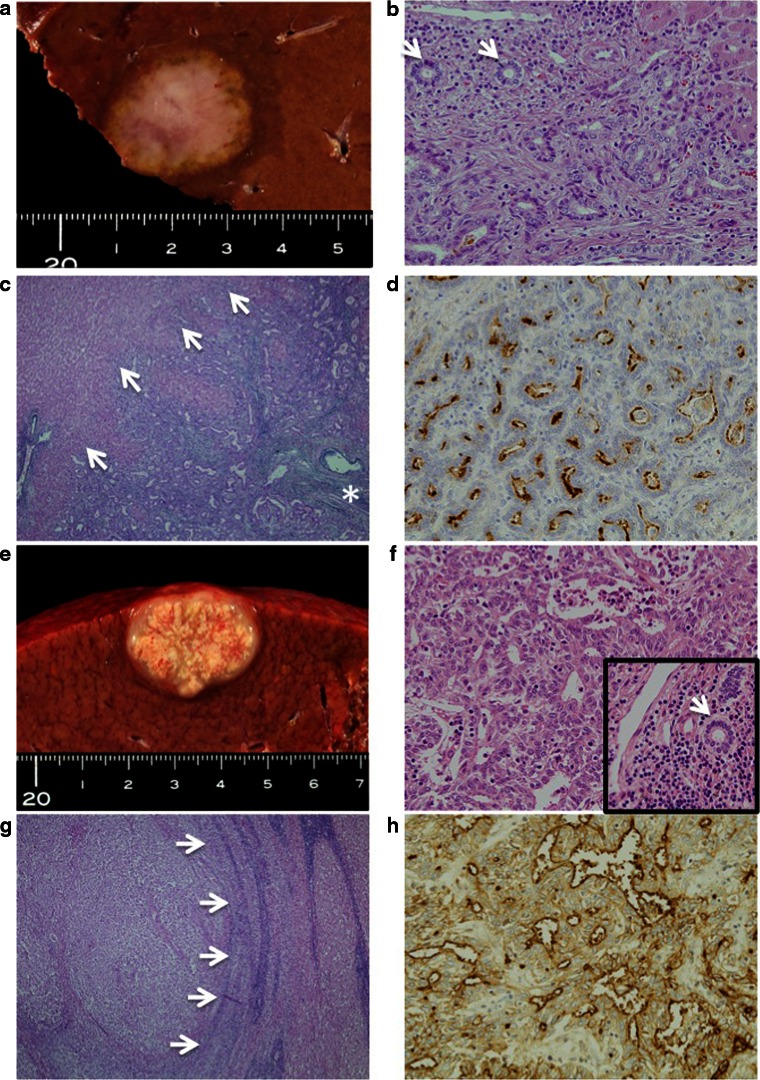
CoCC and ICC showed MF type on macroscopically (**a**) and (**e**). The CoCC cells were proliferated showing small glands, and the size of the small glandular formation was similar or less than the size of normal interlobular ductules (*arrows*) (**b**, ×20). CoCC cells proliferated replacing the hepatocytes of the surrounding hepatic parenchyma (*arrows*), and a remaining portal tract within the tumor (*asterisk*) was seen on VHE staining (**c**, ×4). Immunohistochemically, CoCC cells were membranous positive for EMA (**d**, ×20). The ICC cells were proliferated showing irregular glands, and the size of the glands was larger than the size of normal interlobular ductules (**f**, *arrow in small square*, ×20). The ICC grew compressing the normal hepatocytes (*arrows*, ×10), and no portal tract within the tumor was seen (**g**). Immunohistochemically, ICC cells were cytoplasmic positive staining for epithelial membrane antigen (**h**, ×20)

Pathologically, CoCC cells were defined as small gland-forming cells (Fig. 1). The tumor was composed of small cuboidal cells with round nuclei, basophilic cytoplasm, no nucleoli, and various degrees of fibrous stroma with or without mucin production.^8^,^9^ The size of the small glandular formation was similar or less than the size of normal interlobular ductules.^10^ The CoCC cells sometimes formed antlerlike anastomosing and ductal plate malformation-like patterns.^8^,^15^,^16^ CoCC cells were further confirmed by either positive staining for cytokeratin 19 (CK19), membranous positive staining for mucin core protein 1 (MUC-1), and/or membranous positive staining for epithelial membrane antigen (EMA), but no staining for hepatocyte paraffin 1 (Hep1).^10^,^11^

**Fig. 2:**
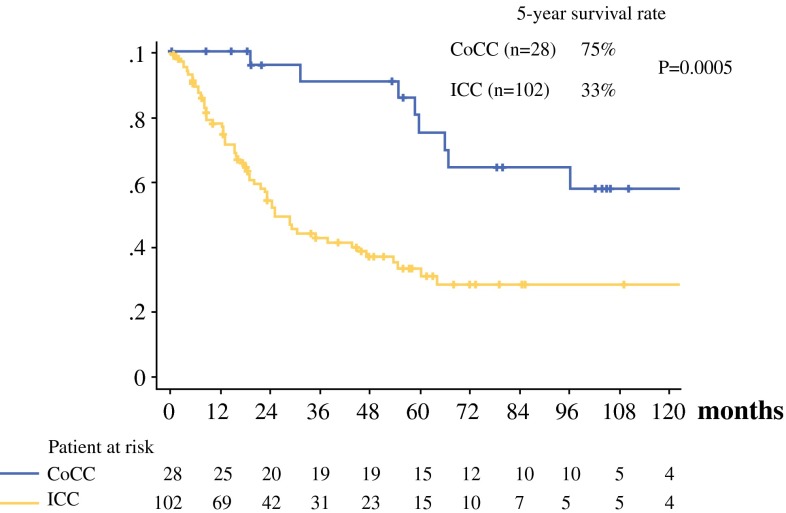
Survival curves of patients with CoCC and ICC who underwent curative surgery

ICC cells were defined as large glandular forming, composed of cuboidal and/or columnar cells with round nuclei, no nucleoli, basophilic cytoplasm, and various degrees of fibrous stroma with or without mucin production (Fig. 2).^8^,^9^,^15^ The size of the glandular formation was larger than the size of normal interlobular ductules.^11^ The ICC cells sometimes formed papillary and solid patterns. ICC cells were confirmed by either positive staining for CK19, cytoplasmic positive staining for MUC-1, and/or cytoplasmic positive staining for EMA, but negative staining for Hep1.^10^,^11^

A pathologist (M.N.) with liver expertise confirmed the diagnoses on the basis of macroscopic, microscopic, and immunohistological findings. When CoCC cells and ICC cells coexisted in the same tumor and the CoCC cells were predominant within the tumor (50 % or more), the tumor was given a diagnosis of CoCC.^8^ When HCC cells coexisted in the same tumor and the HCC cells accounted for 10 % or more of all cells within the tumor, the tumor was diagnosed as combined hepatocellular and cholangiocarcinoma (CHC).^7^,^17^,^18^ Mucus secretion was confirmed by Alcian blue and periodic acid Schiff double staining. Growth pattern at the periphery of the tumor was classified into replacement growth pattern (cancer cell growth replacing the normal hepatocytes) or compressive growth pattern (cancer cell growth compressing the normal hepatocytes).^6^,^8^,^9^,^15^ The number of remaining portal tracts at the periphery within the tumor of each patient was assessed on Victoria blue with hematoxylin and eosin (VHE) staining with 4 × magnification. TNM classifications were determined according to the American Joint Committee on Cancer (AJCC)/International Union Against Cancer (UICC) TNM Classification and Stage Groups for ICC, seventh edition.^19^ Furthermore, another pathologist (NY) with liver expertise confirmed the diagnoses, and all patients were classified according to their new classification for ICC.^15^,^16^

### Follow-Up and Treatment of Patients with Recurrence

After surgery, patients were followed up every 4–12 weeks at the outpatient department of our institution. Ultrasonography or CT was performed once every 3–4 months. Survival duration was defined as the time from hepatectomy to the date of death or last contact. When a solitary CoCC or ICC had recurred in the liver, repeat hepatectomy or radiofrequency ablation was performed. When multiple tumors had recurred in the liver, transarterial chemoembolization (TACE) was performed. If CoCC or ICC recurred in the liver and/or other organs including lymph nodes, systemic chemotherapy or best supportive care was performed.

### Statistical Analysis

Categorical variables were assessed using the Chi square test. Continuous variables were expressed as the median and assessed using the nonparametric Mann–Whitney *U* test. The overall survival rates among the patients were calculated by the Kaplan–Meier method and compared with the log rank test. Statistical significance was indicated by *p* values less than 0.05 (*p* < 0.05). We used JMP software (version 9.0; SAS Institute, Inc., Cary, NC) for statistical analysis.

## Results

### Patient Characteristics

Patient characteristics are shown in Table 1. The number of patients with chronic liver disease was significantly higher in the CoCC group than in the ICC group. In the CoCC group, 8 of 27 patients (30 %) showed abnormal levels of CA19-9, and the number of patients with abnormal levels of CA19-9 was significantly lower in the CoCC group than in the ICC group. The number of patients with high density on arterial CT findings was significantly higher in the CoCC group than in the ICC group. The number of patients who were given a diagnosis of HCC preoperatively was significantly higher in the CoCC group than in the ICC group. The number of patients with surgical procedure, lymph node dissection, and bile duct resection did not differ between groups.

### Tumor Characteristics

Macroscopically, all patients showed the MF type in both groups. The median tumor size was significantly smaller in the CoCC group than that in the ICC group. In the CoCC group, 3 of 29 patients showed intrahepatic metastasis, whereas in the ICC group, 54 of 130 patients showed intrahepatic metastasis. The number of patients with intrahepatic metastasis was significantly lower in the CoCC group than in the ICC group. In the CoCC group, 15 of 29 patients showed portal vein invasion, whereas in the ICC group, 92 of 130 patients showed portal vein invasion. The number of portal vein invasions was significantly lower in the CoCC group than in the ICC group. The number of patients with lymph node metastasis did not differ between the groups. In the CoCC group, 24 of 29 patients showed the replacement growth pattern. On the other hand, in the ICC group, 104 of 130 patients showed compression growth pattern. The number of patients with the replacement growth pattern was significantly higher in the CoCC group than in the ICC group. In the CoCC group, 28 of 29 patients showed portal tracts within the tumor, and the number of patients with the portal tracts within the tumor was significantly higher in the CoCC group than in the ICC group. According to Nakanuma’s classification, 10 of 29 patients with CoCC showed an area with ductal plate malformation like pattern within the tumor.

### Surgical Outcomes

Of 29 patients with CoCC, 28 patients underwent curative surgery, and 1 patient underwent noncurative surgery because of multiple tumors in the remnant liver. No patient died within 30 days after hepatectomy. There were 15 patients who survived for more than 5 years after curative surgery. The survival rate for patients with CoCC who underwent curative surgery was 75 % at 5 years, and the median survival was 63 months (ranging from 0.6 to 183 months). In the ICC group, 102 of 130 patients underwent curative surgery, and 15 patients survived for more than 5 years. There was 1 patient who died due to lactic acidemia on postoperative day 2 because this patient underwent dialysis for the complication of chronic renal failure. The survival rate for 102 patients with ICC who underwent curative surgery was 33 % at 5 years, and median survival was 19 months (ranging from 0.1 to 184 months). The 5-year survival rate was significantly higher in the CoCC group than in the ICC group (*p* = 0.0005, Fig. 2).

### Recurrence

In the CoCC group, tumor recurrence occurred in 16 of 28 patients who underwent curative surgery. The most frequent recurrence site was the liver (Table 2). The recurrence pattern did not differ significantly between groups. Although no lymph node recurrence was seen in the CoCC group, 20 of 69 patients in the ICC group showed lymph node recurrence (*p* = 0.0095). The 5-year recurrence-free survival rate was significantly higher in the CoCC group (41 %) than in the ICC group (26 %, *p* = 0.0408).

**Table 2:** Recurrence patterns

	CoCC (*n* = 16)	ICC (*n* = 69)	*p* value
Liver	12 (75 %)	33 (48 %)	0.09
Liver and other organs	1 (6 %)	11 (16 %)	0.45
Liver and lymph node	0	5 (7 %)	0.58
Liver, other organs, and lymph node	0	3 (4 %)	0.39
Other organ	3 (19 %)	4 (6 %)	0.12
Lymph node	0	8 (12 %)	0.34
Lymph node and other organs	0	4 (6 %)	0.99
Unknown	0	1 (1 %)	

Other organs refers to lung, bone, or peritoneum

*CoCC* cholangiolocellular carcinoma, *ICC* intrahepatic cholangiocarcinoma

### Prognostic Factors

The univariate analysis of prognostic factors for survival with 14 variables (sex, age, chronic hepatitis, ICGR_15_, AFP, CA19-9, surgical procedure, lymph node dissection, tumor size, portal vein invasion, hepatic vein invasion, intrahepatic metastasis, lymph node metastasis, tumor diagnosis) in patients with MF-type ICC and CoCC who underwent curative surgery is summarized in Table 3. The univariate prognostic factors were entered into a multivariate model to identify independent predictors of survival. Multivariate analysis showed CoCC, portal vein invasion, hepatic vein invasion, and intrahepatic metastasis to be significant independent prognostic factors for overall survival in patients with MF-type ICC and CoCC.

**Table 3:** Univariate and multivariate analysis of prognostic factors of patients with MF type ICC and CoCC

	Univariate analysis			Multivariate analysis		
	No.	5-year survival rate (%)	*p* value	Relative risk	95 % CI	*p* value
CA19-9, U/ml						
≤37	59	56	0.0012	0.605	0.344–1.051	0.07
>37	56	28		1		
Portal vein invasion						
Absent	48	52	0.0132	0.549	0.303–0.957	0.0341
Present	82	35		1		
Hepatic vein invasion						
Absent	108	47	0.0003	0.378	0.197–0.752	0.0064
Present	22	0		1		
Intrahepatic metastasis						
Absent	99	51	<0.0001	0.360	0.209–0.629	0.0004
Present	31	19		1		
Tumor diagnosis						
CoCC	28	75	0.0005	0.421	0.185–0.865	0.0175
ICC	102	33		1		

*CI* confidence interval, *CA19*-*9* cancer-associated carbohydrate antigen 19-9, *CoCC* cholangiolocellular carcinoma, *ICC* intrahepatic cholangiocarcinoma

## Discussion

CoCC is a rare type of primary liver cancer, and it was classified as a special type of ICC.^1^–^3^ The pathologic characteristics of CoCC have been described, but surgical outcomes after hepatectomy in patients with CoCC have not been clarified in detail. Komuta et al.^7^ reported that 6 of 30 patients (20 %) with CoCC survived for more than 5 years after hepatectomy. In our present study, 15 of 28 patients (47 %) survived for more than 5 years after curative surgery. The survival rate for patients with CoCC who underwent curative surgery was 75 % at 5 years, and the median survival was 63 months. The 5-year survival rate was significantly higher in the CoCC group than in the ICC group (33 %, *p* = 0.0005). CoCC is rare, but patients with CoCC showed favorable long-term survival after curative surgery.

ICC often invades the portal vein and spreads to the liver as intrahepatic metastasis via the portal invasion. The rate of intrahepatic recurrence after curative surgery is particularly high, and the 5-year survival rate is 30–40 % in patients with the MF type of ICC.^20^–^28^ Several prognostic factors for survival in patients with ICC such as portal vein invasion, tumor number, and serum CA19-9 level are known. Portal invasion and intrahepatic metastasis of ICC are often seen in patients with abnormal levels of CA19-9.^29^ In our present study, the numbers of patients with portal vein invasion, intrahepatic metastasis, and abnormal levels of CA19-9 were significantly lower in the CoCC group than in the ICC group. CoCC was less invasive to the portal vein, as the number of patients with remaining portal tracts within the tumor was significantly higher in the CoCC group than in the ICC group. Therefore, the number of patients with intrahepatic metastasis and abnormal levels of CA19-9 was significantly lower in the CoCC group than in the ICC group. Furthermore, the 5-year overall survival rate and recurrence-free survival rate were significantly higher in the CoCC group than in the ICC group. Moreover, multivariate analysis showed CoCC to be a significant independent prognostic factor for survival in patients with MF-type ICC and CoCC. CoCC is rare, but patients with CoCC showed special characteristics such as favorable long-term surgical outcome because of its less invasive histopathologic characteristics.

Lymph node metastasis is known as one of the important prognostic factors for survival in patients with ICC, and lymph node recurrence after surgery is one of the most intractable situations in patients with ICC.^20^,^21^,^24^–^27^ In the present study, lymph node metastasis tended to be lower in the CoCC group than in the ICC group. Moreover, no lymph node recurrence of CoCC was seen in the present study. Komuta et al.^7^ also reported that no lymph node recurrence was seen in 29 patients with CoCC after surgery. ICC cells spread with various progression patterns along the Glissonean sheath (portal vein invasion, lymphatic invasion, perineural or intraneural invasion, and bile duct invasion) even in its early stage.^30^,^31^ However, CoCC may be less invasive to the lymphatic duct in the Glissonean sheath because the number of patients with remaining portal tracts within the tumor was significantly higher in the CoCC group than in the ICC group.

The replacement growth pattern of CoCC has been reported to be one of its important pathological characteristics.^7^,^8^ Komuta et al.^7^ reported that all patients with CoCC showed tumor cells proliferated as replacing the surrounding normal liver cell cords and had remaining portal tracts within the tumor. Kozaka et al. and Nakanuma et al.^6^,^15^ reported a bile ductular carcinoma that was thought to be derived from hepatic progenitor cells such as CoCC, and bile ductular carcinoma showed replacing growth and portal tracts within the tumor. In our present study, the majority of CoCC cases showed replacement growth patterns and remaining portal tracts within the tumor. These growth patterns of CoCC may correlate with lower invasiveness to the portal vein and lymphatic duct in the portal tract. Therefore, patients with CoCC showed favorable long-term surgical outcome.

CoCC cells often proliferate heterogeneously, CoCC presents ICC-like and HCC-like areas within the tumor, and CoCC is considered to originate from hepatic progenitor or stem cells.^6^,^7^,^11^ Therefore, CoCC is classified as a stem-cell subtype of CHC according to the modified fourth edition of the WHO classification.^9^ On the other hand, CoCC is independent from ICC and is reclassified as a type of primary liver cancer according to the Liver Cancer Study Group of Japan.^8^ However, neither offers immunohistochemical markers for the diagnosis of hepatic progenitor or stem cells. In our present study, CoCC was diagnosed when small gland-forming cells proliferated on pathological findings, and these CoCC cells were further confirmed by membranous positive staining for MUC-1 and/or EMA, because the usefulness of these staining patterns for confirming CoCC cells, normal ductules, or hepatic progenitor cells has been reported.^10^,^11^,^32^ Furthermore, when HCC cells coexisted in the CoCC and the HCC cells accounted for 10 % or less, the tumor was diagnosed as CoCC.^7^ However, when the HCC cells accounted for 10 % or more, the tumor was diagnosed as CHC. These findings are important to differentiate the diagnosis of CoCC and CHC since the surgical outcomes of these 2 entities are quite different.^18^

In conclusion, CoCC is rare, but patients with CoCC had special characteristics with favorable long-term survival due to its less invasive histopathologic characteristics.
